# Influence of Vanadium on the Microstructure of IN718 Alloy by Laser Cladding

**DOI:** 10.3390/ma12233839

**Published:** 2019-11-21

**Authors:** Kun Yang, Hualong Xie, Cong Sun, Xiaofei Zhao, Fei Li

**Affiliations:** 1 Department of Mechanical Engineering and Automation, Northeastern University, Shenyang 110819, China; 1770207@stu.neu.edu.cn (K.Y.); 1510096@stu.neu.edu.cn (C.S.); xiaofeizhao5@gmail.com (X.Z.); 2 Department of Information Science and Engineering, Shenyang University of Technology, Shenyang 110870, China; lifei@sut.edu.cn

**Keywords:** microstructure, element segregation, laves phase, vanadium, laser cladding

## Abstract

A deleterious Laves phase forms in the solidified structure of Inconel 718 (IN718) alloy during laser cladding. However, effective removal methods have not yet been identified. In this study, we first added the IN718 alloy cladding layers with a trace amount of vanadium (V, 0.066 wt.%). Then, we studied the solidification structure of cladding layers using a confocal laser scanning microscope and scanning electron microscopy. The microstructure and Laves phase morphology were investigated. The distribution of niobium (Nb) was observed by experiment as well. We found that V is evenly distributed in dendrites and interdendritic zones. A more refined dendrite structure, reduced second dendrite arm spacing and lower volume fraction of Laves phase were observed in the solidification structure. The results of linear energy-dispersive X-ray spectroscopy (EDS) indicate that the concentration of Nb decreases with an increasing of the distance from the Laves phase. The V-containing sample displayed a relatively slower decreasing tendency. The IN718 alloy sample was harder with the addition of V. In addition, the porosity of the sample decreased compared with the blank sample. The presented findings outline a new method to inhibit the Nb segregation in IN718 alloy during laser cladding, providing reference significance for improving the performance of IN718 alloy samples during actual processing.

## 1. Introduction

**Figure 1:**
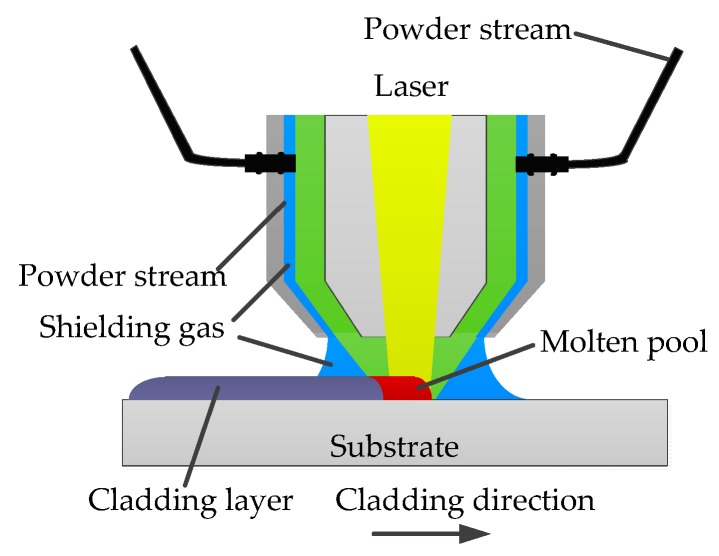
Laser cladding process schematic.

Laser cladding (Figure 1) is a surface modification technology. Compared with traditional surface strengthening technology, laser cladding has high laser beam energy density, high process efficiency, and fast heating and cooling rates [1,2,3]. Nb is the most important element in IN718 alloy. The addition of Nb has a strong solid solution strengthening effect on Ni–Fe–Cr-based austenite and improves the elastic modulus of the alloy. Nb is the elemental basis for the main strengthening phase of IN718 alloy. Meanwhile, one of the most important microscopic characteristics of IN718 alloy is the distribution of Nb-rich Laves phase particles in the matrix during the laser cladding process [4,5]. Laves phase is a hard brittle phase that can provide conditions for nucleation and growth of the cracks under residual stress or other stress [6]. Therefore, the improvement in Nb segregation can benefit microstructure homogeneousness and enhance the performance of IN718 alloy cladding layer.

According to Han et al., IN718 alloy with Mo can reduce the solubility of Nb in the dendrite arm and Laves phases. The addition of Mo transforms the Laves phase morphology from eutectiform to granular and lessens the area of segregation zone around the Laves phase [7]. The effect of the addition of P and B on IN718 alloy as-cast microstructure was studied; the results indicated that the addition of these two alloying elements promoted the formation of a blocky Laves phase. A low melting B-bearing phase enriched in Nb, Mo, and Cr was observed [8]. Xin et al. investigated the effect of Co on precipitation behaviors of IN718 alloy, and the results showed Co was slightly segregated in the dendrite core and markedly increased the solubility of Mo in the dendrite core which resulted in reduced Mo in the residual liquid. Consequently, the Laves phase was retained while precipitation of Mo-depleted gray phase was promoted. The gray phase increased with increasing Co [9]. The effect of Zr on IN718 alloy was investigated as well. The addition of Zr not only inhibited the precipitation of Laves phase at the grain boundary, but also significantly promoted the precipitation of earlobe-like γ′ and γ″ [10]. The addition of W, Ta, and Re other than Nb, was reported to reduce micro-segregation in the fusion zones of IN718 alloy [11]. As a result, adding alloying elements can significantly change the solidification behavior of IN718 alloy.

Vanadium has many desirable physical and chemical properties [12]. It was first used in steel to increase the grain coarsening temperature, which can improve the strength, toughness, and wear resistance of the steel. Afterwards, it was reported that V was found to occupy Al sites in the strengthening phase of Ni-based superalloy. The addition of V also can lead to a significant improvement in the material strength by forming stable nitrides and carbides [13]. 

**Figure 2:**
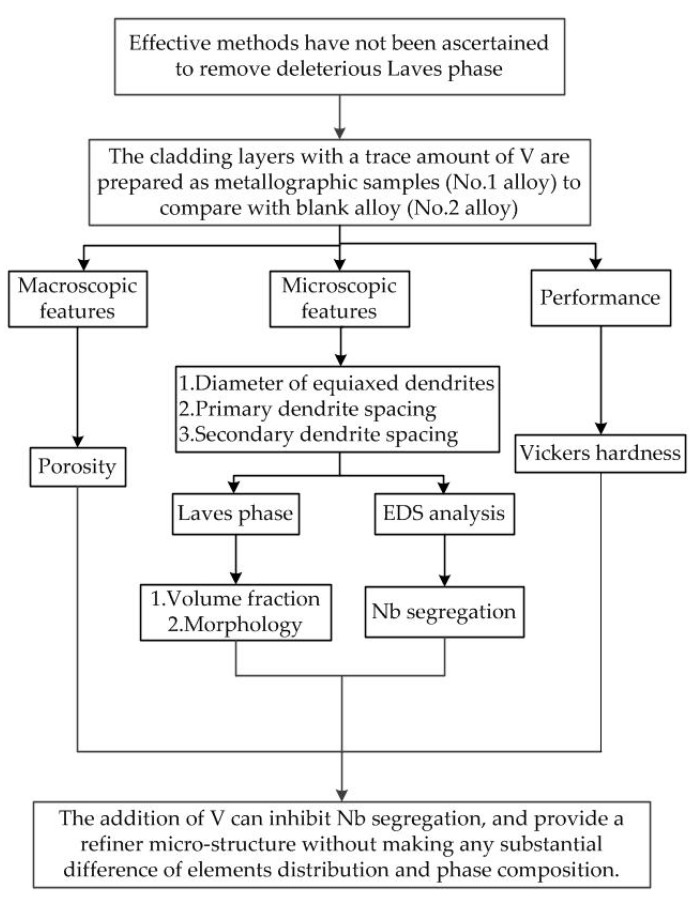
The workflow in this study.

The addition of V appears to positively influence IN718 alloy microstructures. Due to the lack of information of the influence of V on the solidification structure of IN718 alloy during laser cladding, this was our goal in this study. The previous work [7,8,9,10] on the influence of alloying elements on IN718 alloy focused on the solidification and precipitation behaviors. However, macroscopic and microscopic features were not compared between the sample with alloying elements and the blank sample. Furthermore, the addition of other elements can inhibit the formation of Laves phase. Unfortunately, previous studied neglected the quantitative analysis of the Laves phase concentration. Therefore, our focus was to study the influence of V on micro structure, Laves phase formation and performance of IN718 alloy. Figure 2 shows the workflow in this study.

## 2. Materials and Methods

### 2.1. Materials

**Figure 3:**
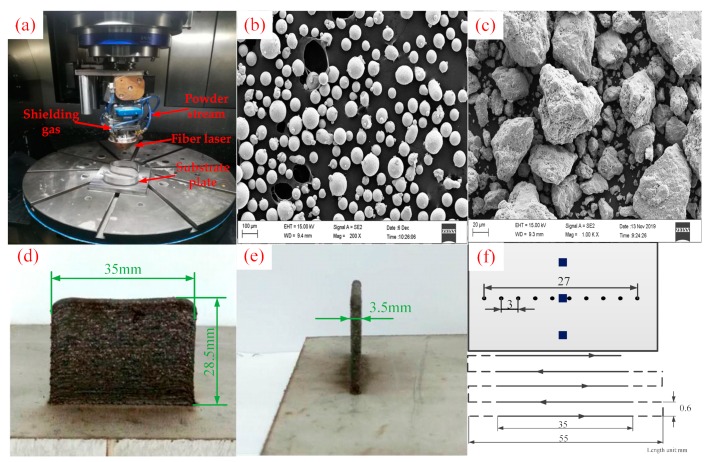
Experimental apparatus: (**a**) laser cladding system, (**b**) morphology of IN718 alloy powder (200×), (**c**) morphology of V powder (1000×), (**d**) main view of the sample, (**e**) left view of the sample, (**f**) Vickers hardness measurement points; secondary dendrite spacing (SDS), primary dendrite spacing (PDS), and diameter of equiaxed dendrites (DED), measurements locations and scan pattern.

The materials in this experiment included spherical IN718 alloy powder (Figure 3a) prepared by the plasma rotation electrode process (PREP), irregular V powder (Figure 3b) prepared by the atomization comminuting process (ACP), and IN718 alloy rolled substrate plate. Both powders had an average diameter of 100 μm. The size of substrate plate was 100 mm × 100 mm × 10 mm.

### 2.2. Laser Cladding Experiment Method

The experiment in this investigation was performed on the adding and subtracting material composite machining center, which is composed of the laser cladding head, powder feeder for laser processing, high purity nitrogen machine, etc., as shown in Figure 3a. The machine was equipped with a fiber laser, which is characterized by high precision, great power, and higher electro-optic conversion efficiency. The laser spot dimensions were 3 mm in length and 1 mm in width. The energy was distributed uniformly over the laser spot due to the property of the fiber laser source. Before the start of the experiment, we ensured that the V powder was evenly distributed in the IN718 alloy powder. To achieve this, the two powders were first stirred in a power agitator for 45 min. Afterwards, the other powders in the powder feeder were emptied to avoid impurities and contamination that would affect the accuracy of the experimental results. Then, 10 cladding layers were cladded on the substrate plate. The process parameters are shown in Table 1. The dimensions of the produced samples are shown in Figure 3d,e. Finally, the cladding layers were cut using wire along the scanning direction to prepare metallographic samples. The solidification structure samples were etched by a Kalling’s etchant (40 mL HCl, 40 mL ethanol, 2 g CuCl_2_). An OLYMPUS-OLS4100 Confocal Laser Scanning Microscope (CLSM, OLYMPUS, Tokyo, Japan) and a Zeiss ULTRA PLUS Scanning Electron Microscope (SEM, Zeiss, Oberkochen, Germany) with a X-Max 50 Energy Dispersive Spectrum (EDS, Oxford, UK) were used to characterize the microstructure and chemical composition. The average diameter of equiaxed dendrites (DED), secondary dendrite spacing (SDS), average size of columnar dendrites’ primary dendrite spacing (PDS) and volume fraction of Laves phase (LPVF) measurements were counted and calculated by Image-Pro Plus6.0 software (Ropers Technologies, Sarasota, FL, USA) using secondary electron micrographs of the etched alloys captured by SEM. Three locations were selected along the height of the sample to measure the DED, SDS, and PDS (Figure 3f). We measured 4–6 dendrites at each location. A total of 15 dendrites were measured for each sample. The micro hardness test was performed using the micro hardness tester with a load (100 mN) and a microdiamond imprint. We selected 10 points along the length of the cross section to measure micro hardness (Figure 3f).

## 3. Results and Discussion

### 3.1. Solidification Structure Characteristics

In this study, we investigated the solidification structure of the V-containing cladding layer (No.1 alloy) from both macroscopic and microscopic aspects. In addition, the results were compared with the samples (No.2 alloy) without the addition of V. The chemical composition comparison between the two alloys is illustrated in Table 2.

**Figure 4:**
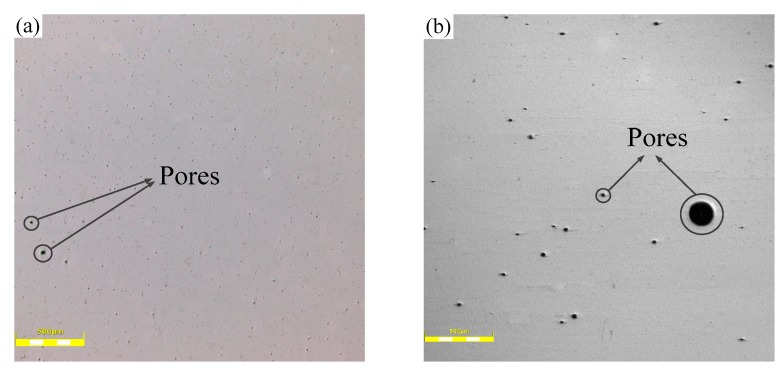
The macroscopic feature of (**a**) No.1 alloy and (**b**) No.2 alloy.

Figure 4 shows the macroscopic feature of the two samples via CLSM (50×). The difference in porosity between the two samples is obvious. The number and the size of pores in the No.1 alloy are much smaller than those of No.2 alloy (Figure 4). The existence of pores degrades the performance of the samples, especially the fatigue property [14]. Therefore, it is reasonable to expect that No.1 alloys would perform better compared to blank alloys.

**Figure 5:**
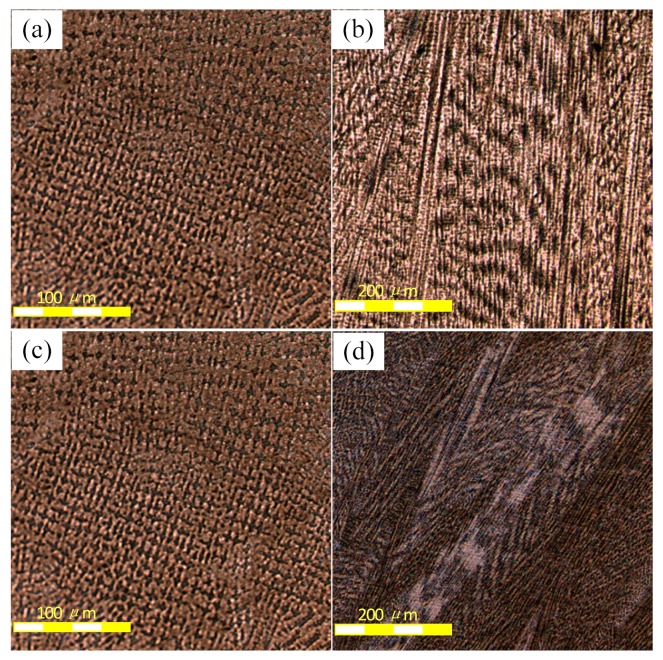
Microstructures of No.1 and No.2 alloy: (**a**) equiaxed dendrite in No.1 alloy; (**b**) columnar dendrite in No.1 alloy; (**c**) equiaxed dendrite in No.2 alloy; (**d**) columnar dendrite in No.2 alloy.

**Figure 6:**
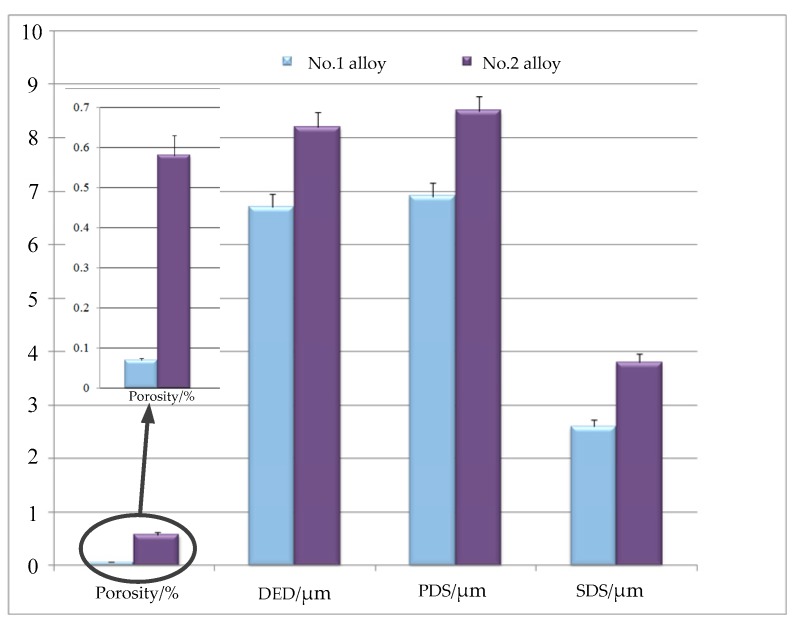
Comparison of macroscopic and microscopic morphology of two alloys.

Figure 5 shows the micro structure of No.1 and No.2 alloys after solidification. The solidification structure of the two samples shows typical dendrite morphology at lower magnifications (200×). The black areas are dendrite, whereas the white areas are the interdendritic precipitation phase. As a result, the average diameter of equiaxed dendrites (DED) in V-containing cladding layers is smaller, as well as the average size of the columnar dendrites’ primary dendrite spacing (PDS) when compared with the blank alloys. To further examine the influence of the addition of V on solidification structure, we measured the secondary dendrite spacing (SDS) of the two alloys. Because this parameter is a key index, it can be used to characterize the microstructure [11]. As shown in Figure 6, the SDS value of the No.1 alloy was 2.6 μm, which is smaller than the 3.8 μm of the No.2 alloy. Consequently, the addition of V can refine the dendrite structure and decrease the secondary dendrite spacing.

The addition of a trace amount of other powder into the original powder may change several parameters and mechanical properties of cladding layers. Generally, a more refined secondary dendrite spacing is desirable. According to Ahmadetal et al. [15], SDS depends on the composition and existence of additive elements, which is used to describe the scale of columnar dendritic structures [16].

Solidification structures with smaller secondary dendrite spacing limit the diffusion range of Nb Hence, decreasing the area ratio of the element segregation regions was more effective to achieve homogenization after heat treatment in No.1 alloy. In summary, the addition of V decreases the porosity in cladding layers and leads to a certain degree of dendrite refinement. Refined solidification and low porosity can enhance the performance of cladding layer.

### 3.2. Influence of V on Element Segregation

**Figure 7:**
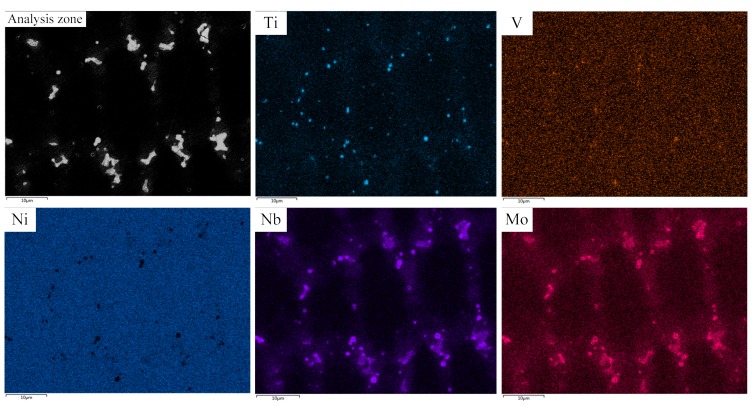
Elements distribution of No.2 alloy.

**Figure 8:**
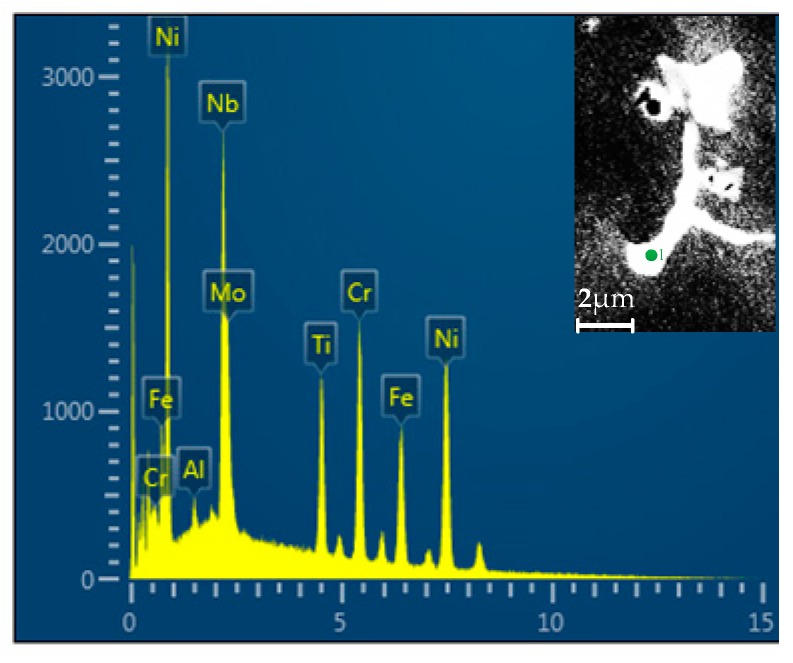
Energy-dispersive X-ray spectroscopy (EDS) data of Laves phase.

In this study, we performed a surface scan analysis on V-containing cladding layers. Figure 7 indicates the distribution of elements. The V-rich area in the analysis zone cannot be found. Conversely, the distribution of V was relatively uniform. In this investigation, V was solid-solved into the austenite matrix, which facilitates the formation of fine carbides at the grain boundaries [17]. These fine V-containing carbide particles create a pinning effect at the austenite grain boundaries, then the grain boundary migration, and the dendritic growth can be hindered [18]. This finding was confirmed by the reduction in the secondary dendrite arm spacing (Figure 6). According to a previous study [19], due to the redistribution property, Nb is prone to causing element segregation during the solidification of IN718 alloy. Detailed energy-dispersive X-ray spectroscopy (EDS) data of the Laves phase is shown in Figure 8. The insert shows an enlarged view of a lumpy Laves phase. The concentration of Nb is the second highest among all elements. Figure 8 also indicates that Nb is abundantly enriched in the interdendritic region. The segregation of Nb was found to be a key factor that controls the formation of Laves phase [20]. Therefore, we discussed whether V can improve Nb segregation. With this goal, we determined the chemical composition of the Laves phase in No.1 and No.2 alloy respectively. The results are listed in Table 3.

**Figure 9:**
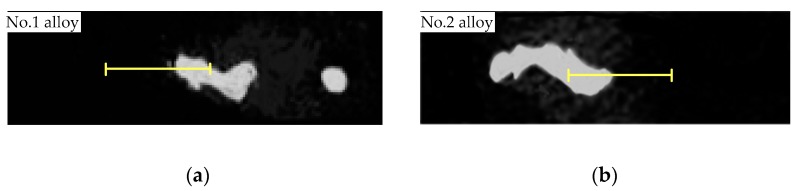
Liner EDS analysis of the Laves phase in (**a**) No.1 alloy and (**b**) No.2 alloy.

**Figure 10:**
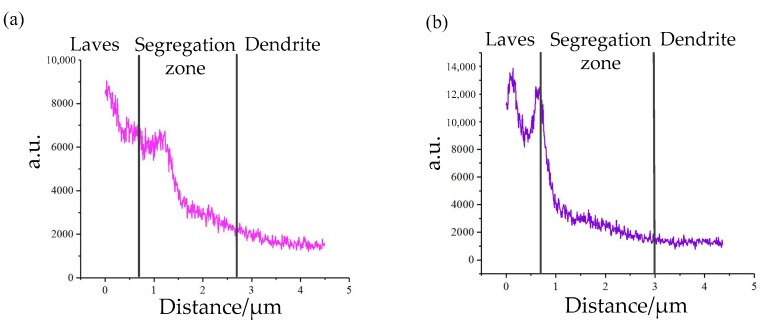
The distribution of Nb in (**a**) No.1 alloy and (**b**) No.2 alloy.

Table 3 shows that the precipitated phase of two alloys have similar elements. However, the Nb concentration in the Laves phase decreases from 26.15 to 21.24 wt.% with the addition of V. This indicates that more Nb is solid-dissolved into the matrix to prepare for strengthening phase precipitation. We concluded that the addition of V improves Nb segregation in IN718 alloy during laser cladding. To further investigate the influence of V on element segregation, we conducted linear EDS analysis on No.1 and No.2 alloys (Figure 9), respectively. The result is shown in Figure 10. The path of the linear EDS passes through the Laves phase, the segregation zone, and the dendrite. Notably, the Nb concentration in the Laves phase of the No.2 alloy fluctuates considerably, which may be caused by the existence of pore.

The results of linear EDS analysis indicated that Nb is heavily enriched in Laves phase. The Nb concentration decreases with increasing distance from the Laves phase. This tendency was observed in both No.1 and No.2 alloys. The difference was that the V-containing sample displayed a slower decreasing tendency. In addition, the average concentration of Nb in the analysis zone of No.1 alloy was less than that of the No.2 alloy, which is consistent with the results in Table 3. All the above results indicate the addition of V decreases the Nb concentration in the Laves phase, and evens out the distribution of Nb in cladding layers.

### 3.3. Influence of V on Laves Phase Formation

**Figure 11:**
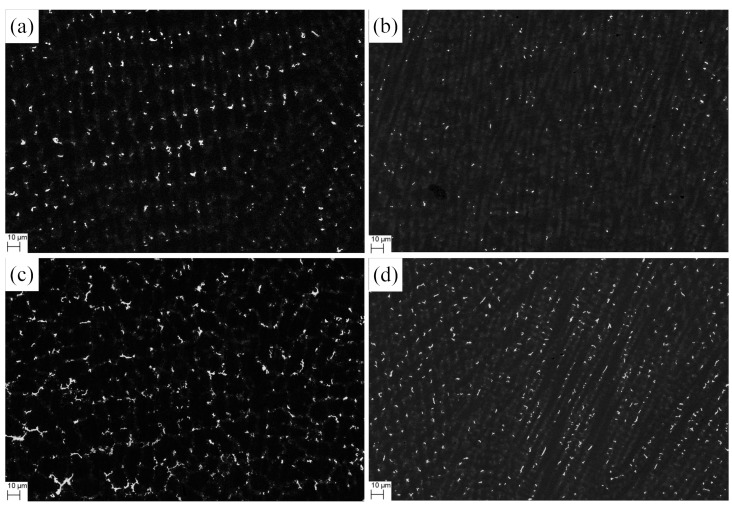
The morphology of Laves phase in the cladding layer: (**a**) in equiaxed interdendritic regions of No.1 alloy; (**b**) in columnar interdendriticregions of No.1 alloy; (**c**) in equiaxed interdendritic regions of No.2 alloy; (**d**) in columnar interdendritic regions of No.2 alloy.

As is stated before, the existence of Laves phase can drastically degrade the performance of IN718 alloy. Under higher magnifications (2000×), a clear island-like segregation zone can be defined, in which the Laves phase particles are distributed (Figure 8). The addition of V can reduce the secondary dendrite spacing and decrease the concentration of Nb in the Laves phase. The microstructure becomes refined, reducing the segregation areas of elements. On this basis, we hypothesized that V can decrease the concentration of Laves phase and modify its morphology. To count the concentration of Nb-rich Laves phase and map its morphology, we conducted electron back scattered diffraction (EBSD) examination on both No.1 and No.2 alloys respectively. The Laves phase distribution of the two alloys in the equiaxial dendrite zones and columnar dendrite zones is shown in Figure 11. The white region represents Laves phase and the austenite appears as black. According to the theory of quantitative metallography [21], the area ratio of the white regions represents the volume fraction of Laves phase in the cladding layers.

Figure 11a,b show that the Laves phase morphology was modified dramatically with the addition of V. In the No.1 alloy, the Laves phase is particle-like. However, the Laves phase that formed in equiaxed interdendritic regions of No.2 alloy is reticular, as displayed in Figure 11c, and in columnar interdendritic region, the Laves phase is rod-like, as shown in Figure 11d. Normally, the particle-like Laves phase is the most desirable morphology feature [22], which could produce the preferred performance in IN718 alloy.

**Figure 12:**
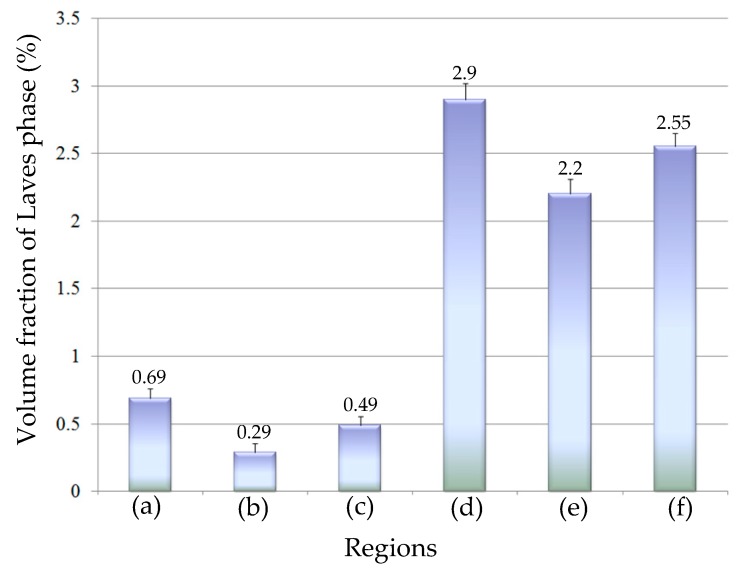
The average volume fraction of Laves phase (LPVF) (**a**) in equiaxed interdendritic regions of No.1 alloy; (**b**) in columnar interdendriticregions of No.1 alloy; (**c**) LPVF of No.1 alloy; (**d**) in equiaxed interdendritic regions of No.2 alloy; (**e**) in columnar interdendriticregions of No.2 alloy; (**f**) LPVF of No.2 alloy.

Figure 12 shows the average volume fraction of Laves phase (LPVF) in equiaxed interdendritic regions and in columnar interdendritic regions of No.1 and No.2 alloys respectively. We found that in the blank sample, the LPVF in equiaxed interdendritic regions was 24% higher than in columnar interdendritic regions, potentially due to the difference in the morphology of the two dendrites. With the addition of V, the difference in LPVF between different regions increased to 58%. Meanwhile, the statistical results indicate that the volume fraction of Laves phase in No.1 alloy decreased by 80% compared with No.2 alloy, from 2.55% to 0.49%. If heat treatment is used to eliminate the Laves phase, the time required for homogenization of the No. 1 alloy would be much shorter than that of No. 2 alloy.

### 3.4. Influence of V on Hardness of Cladding Layer

**Figure 13:**
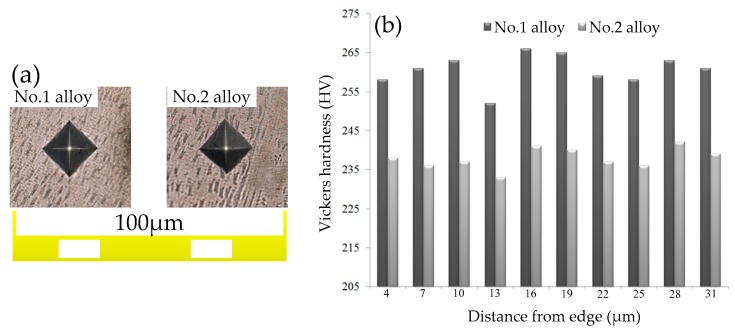
Vickers hardness testing. (**a**) Typical indentation surface morphologies of two alloys; (**b**) comparison of Vickers hardness between No.1 and No.2 alloy.

Hardness is an important performance index used to measure the hardness degree of metal materials. Meanwhile, hardness has been widely used as a preliminary evaluation of the wear resistance of alloy [23]. Therefore the Vickers hardness (HV) values were obtained to tentatively estimate the V-addition influence on the wear resistance of IN718 alloy. Figure 13a depicts the typical indentation surface morphologies of the two alloys showing the smooth and regular rhombus shapes without any cracks or other defects, which indicates the fine metallurgical bonding and a superior relative density of both samples. Figure 13b shows that the average micro hardness of the No.1 alloy was 260.6 HV, whereas that of the No.2 alloy was 237.9 HV. We concluded that with the addition of V, the average micro hardness of the sample increased by 9.5%. According to previous studies [24,25], the Laves phase morphology and volume fraction are the main factors affecting the micro hardness of IN718 alloy. Our comparison of the average micro hardness between No.1 and No.2 alloy supports this finding. To study the dispersion of the micro hardness distribution in the samples, the coefficient of variation (CV) of the experimental data was calculated and compared. The CV of the micro hardness of the No.1 alloy was 0.017, which is slightly higher than that of theNo.2 alloy (0.011). The results illustrate the average micro hardness increases with the addition of a trace of V; however, the micro hardness distribution in the V-containing sample becomes uneven with respect to the blank sample. Based on the empirical correlation between hardness and wear resistance, it is reasonable to expect that the wear resistance of No.1 alloy increases with the addition of V.

The discussion above indicates that the addition of V can inhibit element segregation and change the morphology of the Laves phase, which is similar to adding other alloying elements. However, the decreased of porosity and increased micro hardness in the IN718 alloy sample fabricated by laser cladding was not mentioned in previous studies. Furthermore, a new phase precipitated in the interdendritic region of IN718 alloy with the addition of B or Co, such as B-bearing phase and Mo-depleted gray phase, and the influence on performance is unknown. The addition of V can provide more refined microstructure, without substantially affecting the element distributions and phase compositions.

## 4. Conclusions

In this study, the influence of V on IN718 alloy solidification structure during laser cladding was investigated using experiments. Based on the obtained results our main conclusions were drawn:

(1) With the addition of V, the porosity of cladding layer decrease by 89%, and the average secondary dendrite arm spacing decreased from 3.8 to 2.6 μm, as did the average size of dendrite.

(2) The Nb concentration in the Laves phase of the V-containing sample decreased compared with blank sample. The results of linear EDS indicate that Nb concentration decreases with the increase in the distance from the Laves phase. However, the V-containing sample displayed a slower decreasing tendency.

(3) The morphology of the Laves phase in the V-containing cladding layer was modified dramatically, which changed from rod-like shape to a particle-like feature. The volume fraction of the Laves phase decreased by 80% with the addition of a trace amount of V.

(4) The hardness of the V-containing alloy was higher compared with the blank alloy. However, the distribution of micro hardness was uneven.

Consequently, the addition of V positively influence the microstructure and element segregation of IN718 alloy cladding layers. This investigation provides a new and effective method for inhibiting the formation of Laves phase and enhancing the performance of IN718 samples fabricated by laser cladding.

## Figures and Tables

**Table 1:** Processing parameters of laser cladding.

Parameters	Laser Power (W)	Scanning Speed (mm/s)	Powder Federate (g·min^−1^)	Shield Gas Flow (L·min^−1^)
-	1200	8	18	15

**Table 2:** Chemical composition of two alloys (weight percentage wt.%).

Elements	Ni	Cr	Nb	Mo	Ti	Al	C	Fe	V
**No.1 Alloy**	53.1	18.43	5	3.18	1.06	0.54	0.014	18.61	0.066
**No.2 Alloy**	53.2	18.28	5	3.2	1.08	0.54	0.015	18.64	-

**Table 3:** Chemical composition of Laves phase (wt.%).

Alloy	Ti	Cr	Fe	Ni	Nb	Mo
No.1	1.64	14.14	12.95	45.04	21.24	5
No.2	2.24	13.18	16.98	34.97	26.15	6.28
